# Surrogate markers and survival in women receiving first-line combination anthracycline chemotherapy for advanced breast cancer

**DOI:** 10.1038/sj.bjc.6602858

**Published:** 2005-11-08

**Authors:** A Hackshaw, A Knight, P Barrett-Lee, R Leonard

**Affiliations:** 1 Cancer Research UK & UCL Cancer Trials Centre, Stephenson House, 158-160 North Gower Street, London, NW1 2ND, UK; 2 Evicom Ltd, UK; 3 Velindre Hospital, Cardiff, UK; 4 Singleton Hospital, Swansea, UK

**Keywords:** breast cancer, survival, surrogate markers, metaregression

## Abstract

Surrogate markers may help predict the effects of first-line treatment on survival. This metaregression analysis examines the relationship between several surrogate markers and survival in women with advanced breast cancer after receiving first-line combination anthracycline chemotherapy 5-fluorouracil, adriamycin and cyclophosphamide (FAC) or 5-fluorouracil, epirubicin and cyclophosphamide (FEC) . From a systematic literature review, we identified 42 randomised trials. The surrogate markers were complete or partial tumour response, progressive disease and time to progression. The treatment effect on survival was quantified by the hazard ratio. The treatment effect on each surrogate marker was quantified by the odds ratio (or ratio of median time to progression). The relationship between survival and each surrogate marker was assessed by a weighted linear regression of the hazard ratio against the odds ratio. There was a significant linear association between survival and complete or partial tumour response (*P*<0.001, *R*^2^=34%), complete tumour response (*P*=0.02, *R*^2^=12%), progressive disease (*P*<0.001, *R*^2^=38%) and time to progression (*P*<0.0001, *R*^2^=56%); *R*^2^ is the proportion of the variability in the treatment effect on survival that is explained by the treatment effect on the surrogate marker. Time to progression may be a useful surrogate marker for predicting survival in women receiving first-line anthracycline chemotherapy and could be used to estimate the survival benefit in future trials of first-line chemotherapy compared to FAC or FEC. The other markers, tumour response and progressive disease, were less good.

Survival time is the generally accepted outcome used to assess the overall benefit of treatment for advanced breast cancer. However, demonstration of a survival benefit following first-line chemotherapy can be obscured by the increasing use of effective second and third-line chemotherapeutic agents. Surrogate markers, such as tumour response, may help to predict the effects of first-line treatment on survival. A'Hern *et al*, 1988 used the results of 50 randomised trials of chemotherapy in the treatment of breast cancer and showed that there was a statistically significant relationship between tumour response and survival. Such a relationship has recently been shown in patients with advanced colorectal cancer receiving first-line chemotherapy, though the ability to predict survival for a given tumour response was not as precise as expected (Buyse *et al*, 2000b). We here examine the relationship between several surrogate markers (including tumour response) and survival in women with advanced breast cancer after receiving first-line combination 5-fluorouracil, adriamycin and cyclophosphamide (FAC) or 5-fluorouracil, epirubicin and cyclophosphamide (FEC) chemotherapy in clinical trials.

## METHODS

Assessment of the relationship between survival and surrogate end points is best done when based on data from randomised trials (Buyse and Piedbois, 1996).

### Data

In all, 42 randomised trials were identified from the published literature (Medline 1966–2005) that compared two or more first-line combination therapies in women with metastatic breast cancer. The search criteria included the terms ‘breast’, ‘advanced or metastatic or metastases’, ‘fluorouracil or 5-FU’, ‘cyclophosphamide’, ‘trial or random^*^’ and ‘adriamycin or adriamicin or doxorubicin or epirubicin or epidoxorubicin or anthracycline’. Trials were included in the analyses if they met the following criteria:

All women had metastatic disease (some trials included women with recurrent breast cancer).
Women had received no previous chemotherapy for advanced disease.
If patients had previously been given adjuvant chemotherapy they had to have had clear evidence of relapse and the original therapy could not have included any anthracyclines
One of the treatment regimes included FAC or FEC.

The surrogate markers included in this analysis were complete or partial tumour response, disease progression and time to progression. From each published report the following information was obtained for each treatment group, found directly in the results or by estimation from the illustrations:

- The proportion of patients with a complete and partial tumour response
- The proportion of patients with progressive disease
- The median time to disease progression (months); taken as the time from randomisation (or start of treatment) to the first sign of progression or relapse. There were 9 trials that defined this as the time from randomisation to progression, relapse or death. These were not included in the main analysis but the results are reported separately
- The median survival time (months); taken as the time from randomisation (or start of treatment) to the date of death from any cause

### Statistical methods

The method used here is similar to that described by A'Hern *et al*, 1988. We refer to the FAC or FEC treatment group as Group 2 and the comparison treatments as Group 1. Briefly, the following information (illustrated for complete response) was obtained for each trial and for tumour response and progressive disease:



The odds ratio of having a complete response in Group 1 compared to Group 2 is given by (A × D)/(B × C), but after adding 0.5 to each of the four terms to allow for groups with zero events. These ratios can be used to describe the treatment effect on the surrogate marker. The treatment effect on time to progression was estimated as the median time to progression in Group 1 divided by the median time in Group 2.

The hazard ratio was taken as the median survival time in Group 1 divided by the median time in Group 2, assuming that survival follows an exponential distribution. This is referred to as the treatment effect on survival.

The relationship between the treatment effect on the surrogate marker (odds ratio) and the treatment effect on survival (hazard ratio) was examined using a linear regression, both on a log scale and weighted by the inverse of the variance of the odds ratio. For the regression of survival against time to progression, the number of patients in the study was used as weights. To avoid spurious associations resulting from forcing the regression through the origin (where no treatment effect on the surrogate marker indicates no treatment effect on survival), all regressions contained an intercept term and were of the form log_10_ survival ratio=*a*+*b* × log_10_ odds ratio.

From each regression model, the coefficient of determination (*R*^2^) was obtained; this is the proportion of the variability in the treatment effect on survival that is explained by the treatment effect on the surrogate marker.

It is realised that the method of assessment of tumour response has varied over time and this could affect the proportion of patients with a complete or partial tumour response. However, because the same method of assessment was used for all treatment groups in each trial, it is likely that the odds ratio (which is based on comparing two groups) would not be greatly affected.

## RESULTS

The 42 randomised trials (Table 1) were based on 9163 women and 46 estimates of hazard ratio. In most trials the treatment regimens that were compared to FAC or FEC resulted in a reduction in the proportion of patients with complete or partial tumour responses, an increase in progressive disease and shorter median survival times.

**Figure 1:**
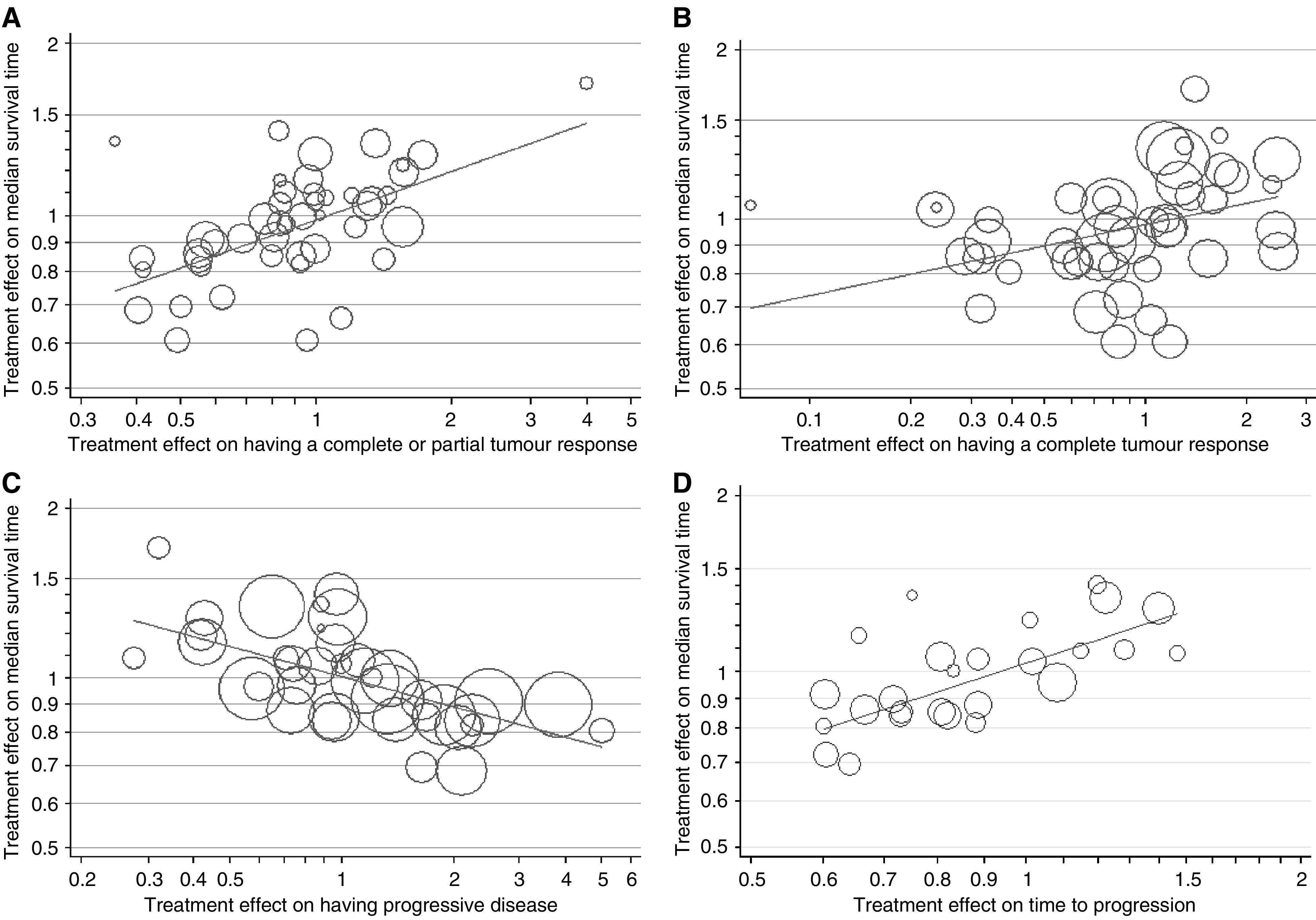
The relationship between the treatment effect on median survival time and each of the four surrogate markers. The regression lines are as follows, with the corresponding *P*-value, coefficient of determination (*R*^2^) and standard error of the regression coefficient (s.e.) in brackets: (**A**) Log_10_ hazard ratio=−0.0081+0.2796 × log_10_ odds ratio for complete/partial response (*P*<0.0001, *R*^2^=34%, s.e.=0.0590), (**B**) Log_10_ hazard ratio=−0.0097+0.1266 × log_10_ odds ratio for complete response (*P*=0.02, *R*^2^=12%, s.e.=0.0521), (**C**) Log_10_ hazard ratio=0.0015–0.1781 × log_10_ odds ratio for progressive disease (*P*<0.0001, *R*^2^=38%, s.e.=0.0380), (**D**) Log_10_ hazard ratio=0.0135+0.5082 × log_10_ ratio of median time to progression (*P*<0.001, *R*^2^=56%, s.e.=0.0928). The size of the symbols is proportional to the inverse of the variance (the weight). For time to progression the size is proportional to the number of patients in the trial.

Figure 1 shows the relationship between the treatment effect on the median survival time (survival ratio) and the treatment effect on tumour response and disease progression (odds ratio). There was a statistically significant linear association between survival and complete or partial tumour response (*P*-value <0.0001); 34% of the variability in the treatment effect on survival can be explained by the treatment effect on tumour response. When the data are restricted to only those patients with a complete response, there was still evidence of a linear association with survival (*P*-value 0.02), though only a small proportion of the variability could be explained (*R*^2^=12%). There was also a relationship with progressive disease (*P*-value<0.0001, *R*^2^=38%) and time to progression (*P*-value <0.0001, *R*^2^=56%); the latter suggesting that a moderately high proportion of the variability in the treatment effect on survival can be explained by the treatment effect on time to progression. The results on time to progression were similar in the 9 trials that included death as an event (regression coefficient 0.4817, *P*-value=0.017, *R*^2^=58%).

There is a possibility that second-line therapies may have obscured the relationships between survival and the surrogate markers. To assess this effect we compared the regression analyses in trials that recruited patients before 1990, when second-line therapies would have been uncommon, to those that recruited in 1990 or later. Table 2 shows the results from this analysis and those from all trials; they are consistent with each other.

Table 3 shows hypothetical examples of two treatments and the predicted effects on survival using the regression equations in Figure 1. For example, if one treatment (A) had a response rate of 30% and a median survival time of 20 months and another (treatment B) was expected to double the response rate to 60%, the estimated median survival using treatment B would be 28 months; an increase in survival of 8 months (Appendix A provides details of the calculation). Similarly, a doubling of the median time to progression was associated with a median survival time that could be 9 months greater.

## DISCUSSION

These results suggest that tumour response and progressive disease are both associated with survival in women receiving first-line FAC or FEC chemotherapy for advanced breast cancer, but the best surrogate marker is time to progression. The strength of the association was only modest for tumour response (*R*^2^=34%) and progressive disease (*R*^2^=38%), but stronger for time to progression (*R*^2^=56%).

The conclusion for tumour response is similar to that reported by A'Hern *et al*, 1988 whose analysis was based on all chemotherapy trials published by 1986. In that analysis an estimated 37% of the variability in survival was explained by variation in tumour response (compared to our estimate of 34%). Our analysis differs to that by A'Hern *et al*, 1988 for several reasons – only 10 of the 42 trials in our analysis could have been included; we only included trials that included FAC/FEC first-line therapies; several surrogate markers were assessed here; and we used a different model to quantify the association between survival and each surrogate marker (we used linear relationships that were not forced to go through the origin thereby avoiding possible spurious associations – A'Hern *et al*, 1988 used a quadratic model that was forced through the origin).

The appeal of a perfect surrogate marker is that if it can be measured earlier than a ‘true’ end point (such as survival) then a trial would require less time spent on following-up patients before a conclusion can be made about the treatment being tested. Furthermore, if one is interested in assessing a first-line therapy then the effect on survival may be obscured if patients are given second- and third-line therapies; the advantage of using a surrogate marker is that it could be measured before these subsequent therapies are administered. Several investigators have discussed various approaches to determine the usefulness of proposed surrogates. Buyse and Molenberghs, 1998 introduce the concept of ‘relative effect’. This compares the treatment effect on survival with the treatment effect on the surrogate marker. The relative effect is simply the slope of the regression line from a regression analysis. A perfect surrogate would have a relative effect of 1. In our analyses the relative effects were small for complete/partial response (0.28) and progressive disease (0.18) but greater for time to progression (0.51). However, a marker could still be useful as a surrogate if it predicts worthwhile changes in the true end point, such as survival. Our results indicate that this may be so (Table 3).

Buyse *et al* (2000a) suggest evaluating surrogacy by estimating two coefficients of determination; *R*^2^_trial_ based on data from the trials and the *R*^2^_indiv_ based on individual patients. A marker would be called ‘trial-level’ valid if *R*^2^_trial_ is close to one and ‘individual-level’ valid if *R*^2^_indiv_ is close to one. The latter would indicate the ability for a marker to predict survival for an individual patient. Furthermore, a large *R*^2^_indiv_ indicates that the surrogate is causally linked to the true end point, an observation that confirms that a surrogate is highly effective. In an example of treating advanced ovarian cancer (Buyse *et al*, 2000a) individual patient data were available so both *R*^2^ values could be estimated. Survival was the true end point and time to progression was the proposed surrogate marker. They found that *R*^2^_trial_=0.94 and *R*^2^_indiv_=0.89, both sufficiently high to conclude that time to progression could be used as a surrogate. In our analyses we did not have individual patient data so were unable to estimate *R*^2^_indiv_. Our estimates for *R*^2^_trial_ were only modest for tumour response (34%) and progressive disease (38%) but greater for time to progression (56%).

There are limitations to our analysis. First, although this analysis was restricted to randomised trials (thereby minimising some biases associated with similar analyses of surrogate markers (Buyse and Piedbois, 1996), it was based on performing regressions using summary data, namely odds ratios and survival ratios. The ability to predict survival from a surrogate marker for an *individual patient* will therefore be limited (Buyse and Piedbois, 1996). Analyses of these trials using individual patient data would provide more precise estimates of the predictive ability of these markers on survival. Second, it was not possible to assess the effect of second-line therapies in patients whose disease progressed during the course of the trials; such therapies may also have had an affect on survival. For instance, a trial by Nabholtz *et al* (1999) showed that patients with advanced breast cancer may benefit in terms of survival from more effective second-line therapy. All patients in this trial had already received first-line anthracycline chemotherapy for metastatic cancer and were randomised to receive either docetaxel or mitomycin plus vinblastine; survival was longer in the docetaxel group (11.4 *vs* 8.7 months). However, our analysis of trials that recruited patients before 1990, when second-line therapies were less likely to have been used, gave similar results to those published after 1990 (Table 2).

Despite these limitations the results may be useful when determining the efficacy of first-line treatments for advanced breast cancer that use anthracyclines. With the increasing use of effective second and third-line chemotherapy in breast cancer this type of analysis offers a means of comparing new first-line chemotherapy treatments to first-line anthracycline combination therapies without the effect being masked by second or third line therapies.

## Figures and Tables

**Table 1:** Selected characteristics of the trials used in the analysis

			**Treatment arms^a^**		
**Trial (first author & reference)**	**Country**	**Year of patient recruitment^b^**	**Group 1**	**Group 2**	**No. of patients randomised**
Muss *et al* (1978)	USA	1976–1976	FMC+VP	FAC+VP	175
Fritze *et al* (1982)	Germany	1975	FAC+VM+C. parvum	FAC+VM	156
Smalley *et al* (1983)	USA	1974–1977	FMC+VP	FAC	362
Steiner *et al* (1983)	USA	1977–1980	FAC+MV	FAC+M	119
Tormey *et al* (1984)	USA	1974–	(a) FMC+VPc	FAC+VP	396
			(b) FMC+VPd		
Vogel *et al* (1984)	USA	<1982	FAC+VM, Leucovorin, Cytosine arabinside	FAC	187
Boccardo *et al* (1985)	Italy	1978–1982	FAC+Tamoxifen	FAC+MV	81
Cummings *et al* (1985)	USA	1978–1979	FMC+P	FAC	177
Carpenter *et al* (1986)	USA	1978–1982	FAC+Levamisole	FAC	105
Aisner *et al* (1987)	USA	1976–1980	(a) FMC	FAC	432
			(b) FAC+VP		
Falkson *et al* (1987)	USA, S. Africa	1968–1983	FAC+oophorectomy	FAC	86
Hortobagyi *et al* (1987a)	USA	1978–1981	High dose FAC+protected environment	Low dose FAC+ambulatory care	63
Hortobagyi *et al* (1987b)	USA	1979–1980	FAC+vaccine	FAC	133
Bennett *et al* (1988)	USA	1983–1985	FNC	FAC	333
French Epirubicin Study Group (1988)	France	1982–1984	FEC	FAC	263
Lopez *et al* (1989)	Italy	1983–1985	FEC	FAC	102
Falkson *et al* (1991) (B122)	USA, S. Africa	1972–	FMC	FAC	78
Falkson *et al* (1991) (B141)	USA	1974–	FMC+AV, dibromodulcitol	FAC	94
French Epirubicin Study Group (1991)	France	<1990	(a) FEC (75 mg/m2)e	FEC (50 mg/m2)e	412
			(b) E (75 mg/m2)e		
Falkson *et al* (1992)	S. Africa	<1992	mitomycin C+PA	FAC	34
Speyer *et al* (1992)	USA	1984–1989	ICRF-187+ FAC	FAC	150
Ejlertsen *et al* (1993)	Denmark	1986–1989	FEC 18 months	FEC 6 months	359
Paridaens *et al* (1993)	Belgium	1983–1987	FAC+ ethinylestradiol	FAC	165
Pouillart *et al* (1994)	France	1983–1984	FNC	FAC	142
Alonso *et al* (1995)	Spain	1988–1991	FNC	FAC	100
Pavesi *et al* (1995)	Italy	1987–1989	FNC	FEC	152
Pierga *et al* (1995)	France	1990–1993	FAC (15 mg/m2)e	FAC (50 mg/m2)e	258
Conte *et al* (1996)	Italy	1985–1990	FEC+D	FEC	258
Pfeiffer *et al* (1996)	Denmark	1985	FEC+Concurrent Tamoxifen	FEC+Sequential Tamoxifen	273
Stewart *et al* (1997)	Canada	1982–1988	FNC	FAC	249
Esteban *et al* (1999)	Spain	1987–1993	FNC	FEC	151
French Epirubicin Study Group (2000)	France	1987–1994	FEC (100 mg/m2)e then FEC (50 mg/m2)e	FEC (75 mg/m2)e	417
			FEC (100 mg/m2)e		
Pacini *et al* (2000)	Italy	1991–1996	EM±lonidamine	FEC±lonidamine	326
Riccardi *et al* (2000)	Italy	1995–1997	FEC (120 mg/m2)e	FEC (60 mg/m2)e	74
Sledge *et al* (2000)	USA	1988–1992	FAC+tamoxifen, fluoxymesterone	FAC	231
Ackland *et al* (2001)	Australia	1990–1992	FMC	FEC	460
Hori *et al* (2001)	Japan	1993–1996	Doxifluridine, C, ‘PA’	FAC+‘PA’	99
Jassem *et al* (2001)	E. Europe, Israel, Russia	<2000	A, Paclitaxel	FAC	267
Mackey *et al* (2002)	USA	1998–1999	Docetaxel,AC	FAC	484
Heidemann *et al* (2002)	Germany	1992–1997	N	FEC	260
Parnes *et al* (2003)	USA	1991–1995	FAC+leucovorin	FAC	241
Zielinski *et al* (2005)	International	1999–2002	Gemcitabine, Paclitaxel, E	FEC	259

a F (5-fluorouracil); A (adriamycin/doxorubicin); C (cyclophosphamide); E (epirubicin); M (methotrexate); V (vincristine); P (prednisone); N (Novantrone/mitoxantrone); ‘PA’ (medroxyprogesterone acetate); D (diethulstillbestrol).

b If not reported, it is taken to be the year before the article was published.

^c^Therapy given continuously.

^d^Therapy given intermittently.

^e^The dose of epirubicin or doxorubicin is given in brackets.

**Table 2:** Comparison of regression analyses in trials that recruited patients before 1990 (when second-line therapies were not commonly used) and after 1990

**Surrogate marker; last year of patient recruitment**	**Number of studies**	**Slope from regression line**	***R* ^2a^**	**P-value from regression analysis**
*Complete/partial response*				
<1990	29	0.28	26%	0.004
1990+	17	0.24	41%	0.005
All	46	0.28	34%	<0.001
				
*Complete response*				
<1990	29	0.09	5%	0.24
1990+	16	0.16	36%	0.01
All	45	0.13	12%	0.02
				
*Progressive disease*				
<1990	21	−0.26	39%	0.002
1990+	17	−0.14	45%	0.003
All	38	−0.18	38%	<0.0001
				
*Time to progression*				
<1990	17	0.58	67%	<0.0001
1990+	9	0.40	41%	0.06
All	26	0.51	56%	<0.0001

a *R*^2^ is the coefficient of determination (the percentage of variability in survival explained by the surrogate marker).

A test comparing the regression slopes (<1990 *vs* 1990+) yielded *P*-values that were not statistically significant – complete/partial response *P*=0.37; complete response *P*=0.26; progressive disease *P*=0.06; time to progression *P*=0.15.

**Table 3:** Two hypothetical treatments (A and B)

**Percentage of patients with a complete/partial tumour response**	**Median survival, months (95% CI)**	**Percentage of patients with progressive disease**	**Median survival, months (95% CI)**	**Median time to progression (months)**	**Median survival, months (95% CI)**
On treatment A		On treatment A		On treatment A	
30	20	12	20	10	20
					
On treatment B		On treatment B		On treatment B	
40	22 (20–24)	10	21 (20–22)	12	23 (21–24)
50	25 (22–28)	8	22 (20–23)	16	26 (23–29)
60	28 (24–33)	6	23 (21–25)	20	29 (25–34)

The estimated effect on survival using treatment B compared to A based on arbitrary estimates of tumour response and disease progression, and the regression analyses in Figure 1.

CI, confidence interval (based on the 95% CI of the predicted mean value in the regression analysis).

## Appendix A

### Example of estimating the effect on survival using two hypothetical treatments

If treatment A has a response rate of 30% and a median survival time of 20 months and treatment B has a response rate of 60%, the estimated survival time for B is 29 months, obtained as follows:

The odds ratio for response (Treatment B compared to Treatment A) is:
The ratio of survival times is estimated using the appropriate regression equation in Figure 1 : log_10_ hazard ratio=−0.0081+0.2796 × log_10_ [odds ratio for complete/partial response] log_10_ hazard ratio=−0.0081+0.2796 × log_10_ [3.5] log_10_ hazard ratio=0.1440
The ratio of the log survival ratios is:

The estimated median survival in Treatment B is: 20 × 10^0.1440^, which is 28 months.
